# Increased duration of mechanical ventilation is associated with decreased diaphragmatic force: a prospective observational study

**DOI:** 10.1186/cc9094

**Published:** 2010-07-01

**Authors:** Greet Hermans, Anouk Agten, Dries Testelmans, Marc Decramer, Ghislaine Gayan-Ramirez

**Affiliations:** 1 Medical Intensive Care Unit, General Internal Medicine, University Hospitals Leuven, Herestraat 49, B3000 Leuven, Belgium; 2 Respiratory Muscle Research Unit, Laboratory of Pneumology and Respiratory Division, Katholieke Universiteit Leuven, Herestraat 49, B3000 Leuven, Belgium

## Abstract

**Introduction:**

Respiratory muscle weakness is an important risk factor for delayed weaning. Animal data show that mechanical ventilation itself can cause atrophy and weakness of the diaphragm, called ventilator-induced diaphragmatic dysfunction (VIDD). Transdiaphragmatic pressure after magnetic stimulation (TwPdi BAMPS) allows evaluation of diaphragm strength. We aimed to evaluate the repeatability of TwPdi BAMPS in critically ill, mechanically ventilated patients and to describe the relation between TwPdi and the duration of mechanical ventilation.

**Methods:**

This was a prospective observational study in critically ill and mechanically ventilated patients, admitted to the medical intensive care unit of a university hospital. Nineteen measurements were made in a total of 10 patients at various intervals after starting mechanical ventilation. In seven patients, measurements were made on two or more occasions, with a minimum interval of 24 hours.

**Results:**

The TwPdi was 11.5 ± 3.9 cm H_2_O (mean ± SD), indicating severe respiratory muscle weakness. The between-occasion coefficient of variation of TwPdi was 9.7%, comparable with data from healthy volunteers. Increasing duration of mechanical ventilation was associated with a logarithmic decline in TwPdi (R = 0.69; *P *= 0.038). This association was also found for cumulative time on pressure control (R = 0.71; *P *= 0.03) and pressure-support ventilation (*P *= 0.05; R = 0.66) separately, as well as for cumulative dose of propofol (R = 0.66; *P *= 0.05) and piritramide (R = 0.79; *P *= 0.01).

**Conclusions:**

Duration of mechanical ventilation is associated with a logarithmic decline in diaphragmatic force, which is compatible with the concept of VIDD. The observed decline may also be due to other potentially contributing factors such as sedatives/analgesics, sepsis, or others.

## Introduction

Weaning from mechanical ventilation is an important and time-consuming process in critically ill patients. Weaning comprises approximately 40% of the time spent on the ventilator [1]. Although several factors may contribute to delayed weaning, a major determinant appears to be respiratory muscle weakness [2]. The most frequent causes of muscle weakness in critically ill patient, which may affect both limbs and respiratory muscles, are critical illness polyneuropathy and myopathy. Many potential risk factors hereof have been described in several prospective trials [3-9]. All of these studies, however, focused on peripheral muscle strength. Limited data are available specifically addressing the respiratory component of muscle weakness, suggesting septic shock to be a strong predictor [2].

Extensive animal data also suggest that controlled mechanical ventilation (CMV) itself, during which the diaphragm is inactive, may cause atrophy of the diaphragm and decreased force-generating capacity *in vitro *and *in vivo *[10-16]. This occurs in a time-dependent way, as early as 12 h after starting CMV [11]. This phenomenon has been called ventilator-induced diaphragmatic dysfunction (VIDD). Although CMV is not the preferred mode of ventilation in the ICU, it is necessary in particular situations, such as during the use of neuromuscular blockade, in attempts to minimize oxygen consumption, central neurologic problems, and so forth, and therefore still is used in the ICU [17]. If VIDD also occurs in humans, it may therefore have important clinical impact. Brain-dead patients undergoing mechanical ventilation for 18 to 69 h indeed showed atrophy of the diaphragm [18]. A reliable tool to measure respiratory muscle force is essential to guide further research concerning causes and consequences of respiratory muscle weakness and potential therapies aimed at preserving respiratory muscle force in critically ill patients.

Recently, a method of measuring diaphragmatic force was introduced by Watson [19] in critically ill patients. This involves stimulation of both phrenic nerves at the anterior side of the neck by using two magnetic coils, called bilateral anterior magnetic phrenic nerve stimulation (BAMPS). The resulting diaphragmatic contraction is registered by using two balloon catheters positioned in the esophagus and stomach. Measuring twitch transdiaphragmatic pressure appeared feasible in critically ill patients, although not all patients can be measured. Critically ill patients had significantly lower diaphragmatic force compared with healthy controls. Later, the same technique was used by Laghi [20], who confirmed the reduced diaphragmatic force in patients ready to be weaned.

The purpose of the present study was to evaluate the repeatability of BAMPS TwPdi on different occasions in critically ill and mechanically ventilated patients. We also aimed to examine whether TwPdi would decrease with increasing duration of mechanical ventilation.

## Materials and methods

### Patients

Patients were eligible if they were admitted between March 2007 and October 2008 to the medical intensive care unit, which is a 17-bed ICU of a tertiary center with approximately 750 admissions yearly. Inclusion criteria consisted of intubation and mechanical ventilation, hemodynamic stability, and stable respiratory status with a positive end-expiratory pressure (PEEP) ≤7 cm H_2_O. Contraindications were those related to magnetic stimulation (pacemaker or implantable cardioverter-defibrillator, prosthetic valve, cervical implants, cervical trauma), contraindication for gastric/esophageal balloon placement (coagulation disorders, low platelet count, gastric or esophageal pathology), factors possibly interfering with correct pressure measurements (multiple-functioning chest drains, severe abdominal infections, recent major abdominal surgery, agitation, bronchospasm), age younger than 18 years, pregnancy, and having received neuromuscular blocking agents in the past 24 h.

Mechanical ventilators used were Dräger, EvitaXL, and Dräger, Evita4. During mechanical ventilation, the need for analgesics and sedatives was daily evaluated and titrated by the treating physician to the minimum needed, aiming at a sedation agitation score [21] of 3 to 4. Informed consent was obtained from the patients or their relatives. The study was approved by the local ethics committee.

### Measurement of diaphragmatic force

We measured twitch transdiaphragmatic pressure (TwPdi) by using bilateral anterior magnetic phrenic nerve stimulation (BAMPS), as described previously [19]. In brief, the phrenic nerves were stimulated bilaterally from the anterior approach, at the posterior border of the sternocleidomastoid muscle, at the level of the cricoids, by using two figure-of-eight 45-mm magnetic coils (Magstim, Dyfed, Wales) and a bistim (Magstim, Dyfed, Wales). A custom-built, two-way occlusion valve was used to create isometric conditions during stimulation. Esophageal and abdominal pressure changes were measured by using balloon catheters (UK Medical, Sheffield, UK) inserted through the nose after local anesthesia. The gastric balloons were filled with 2 ml of air, and the esophageal balloons contained 0.5 ml of air. To verify correct positioning of the abdominal catheter, abdominal compression was applied. The position of the esophageal catheter was confirmed to be correct if the end-expiratory pressure was near the PEEP level applied, and if inspiration against the closed airway resulted in similar pressure changes on the esophageal and tracheal tracings. Tracheal, abdominal (TwPabd), and esophageal pressures (TwPes) were measured by using Validyne MP45 transducers, 250 cm H_2_O, connected to a custom-built carrier amplifier. Tracheal pressure was measure at the proximal end of the endotracheal tube. Biopac MP150 (Cerom, Paris, France) was used as the data-acquisition system. Patients were left breathing quietly for 20 min after balloon placement. After determining the optimal position of the coils, at least three stimulations were performed at 100% of maximal output. To evaluate supramaximality, patients were also stimulated at 70%, 90%, and, if necessary, at 95%. All measurements were performed with the head end in 30-degree upward position. Between two stimulations, at least a 30-sec pause was obtained to avoid superposition. To evaluate repeatability of the measurement, if possible, patients were measured on two occasions as close together as technically feasible and according to the patients' clinical status, but with a minimal time interval of 24 h.

### Analysis of signals

**Figure 1:**
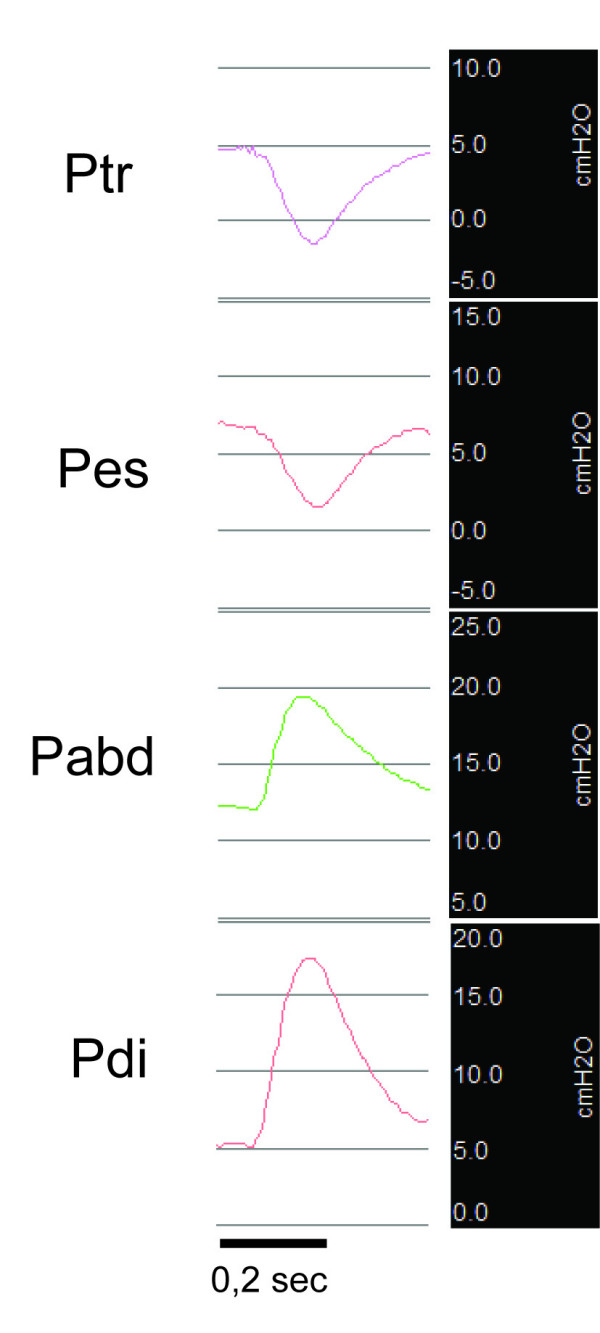
**Tracheal, esophageal, and abdominal pressure tracings on bilateral magnetic stimulation**. Ptr, tracheal pressure; Pes, esophageal pressure; Pabd, abdominal pressure; Pdi, transdiaphragmatic pressure (which was obtained by electronic subtraction of Pabd and Pes).

Individual abdominal and esophageal pressure signals were accepted for analysis if they were timed at end expiration with no major cardiac artefact, if stable and physiologically acceptable end-expiratory pressure was present, and if active abdominal muscle contraction or esophageal contraction during the stimulation was absent. TwPes and Twabd were defined as the maximal excursion of the esophageal and abdominal tracing, respectively, on stimulation from the value immediately before stimulation (Figure 1). TwPdi was calculated as the difference between TwPabd and TwPes. The mean value of at least three signals was made to determine TwPdi on a given occasion.

### Data analysis

Mean values of TwPdi, TwPabd, and TwPes in the total case series were calculated by using the mean value for patients that were measured on more than one occasion. The repeatability of measurement on the same occasion was evaluated by calculating the within-occasion coefficient of variation. Repeatability on different occasions was calculated by using data from patients who were measured on at least two different occasions and by determining the between-occasion coefficient of variation. The relation between TwPdi and duration of mechanical ventilation was evaluated by using regression analysis and applying the logarithmic model.

## Results

### Patient recruitment and characteristics

**Figure 2:**
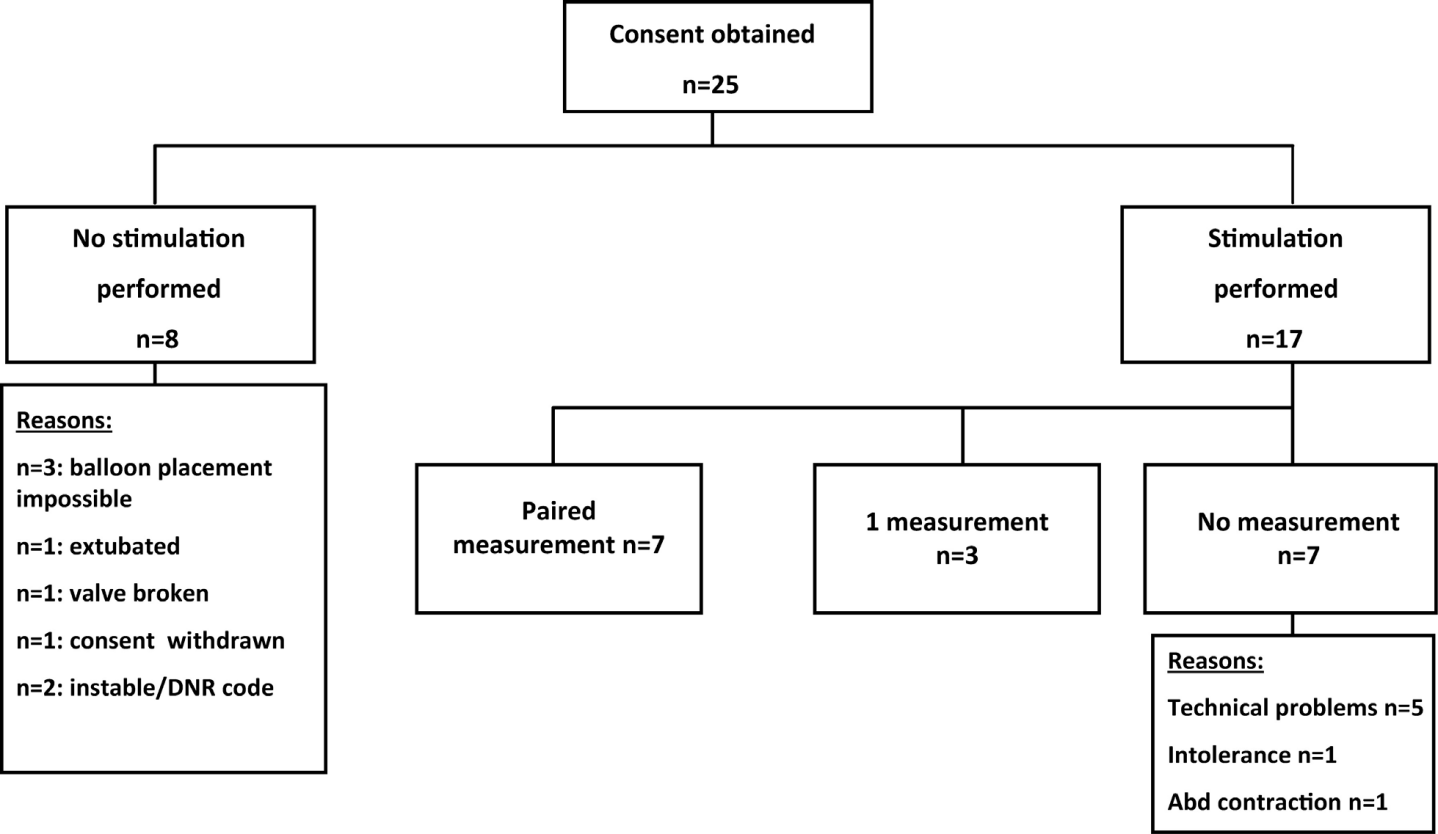
**Patient disposition**.

Informed consent was obtained in 25 patients (Figure 2). In eight of these patients, no stimulation was performed. Reasons were withdrawal of consent in one, inability to position gastric/esophageal catheters in three, technical problems with the valve in one, evolution of the medical condition by the time of the planned measurement, interfering with the planned measurement in three (moribundus, *n *= 1; unstable, *n *= 1; extubated, *n *= 1). In seven of 17 remaining patients, stimulation was performed on at least two occasions with a mean interval of 51.4 ± 35.1 (SD) h between measurements. One of these patients appeared to have unilateral diaphragm paralysis after surgery, confirmed by a phrenic nerve-conduction study, and another patient was measured on 4 consecutive days. In three other patients, measurements were made on a single occasion. In the remaining seven patients, no TwPdi values are available because of intolerance, technical problems, or active abdominal contraction during the experiment.

The reason for admission to ICU and the demographic data of the patients are shown in Table 1. The age ranged from 35 to 78 years. Measurements were made between 2 to 30 days after admission to ICU. All but one patient had sepsis, and except for one patient, all of these received vasopressors. Only one patient received renal-replacement therapy. Three patients were treated with aminoglycosides, and half of the patients received at least one bolus of neuromuscular blocking agents. All measurements were made at a PEEP level of between 5 and 7 cm H_2_O. Patients that were measured on more than one occasion were measured at the same PEEP level. Supramaximal stimulation was reached in 13 of 19 TwPdi measurements; in two of 19, supramaximality was not reached; and in the remaining four of 19, insufficient data are available to evaluate supramaximality.

**Table 1:** Demographic data of patients measured

Patient number	1	2	3	4	5	6	7	8	9	10
Reason for admission	Intestinal perforation and septic shock after redo gastric banding	Cutaneous T-cell lymphoma, generalized weakness	Spondylodiscitis with septic shock	Urosepsis with septic shock	Atrial fibrillation after pneumectomy	Surgical repair of ruptured abdominal aneurysm	Thrombotic, thrombocytopenic purpura	Pyelonephritis with septic shock	Peritonitis	Limbic encephalitis
APACHE II	19	11	19	15	14	23	32	28	9	12
Days after admission	16	17	11	2	11	6	30	8	21	4
Number of measurements	2	1	1	2	2	2	2	1	2	4
TwPdi (cm H_2_O)	12.1	8.3	9.1	12.9	9.1	15.4	8.4	8.9	8.9	18.7
Supramaximal stimulation	Yes	Yes	NA	No	Yes	Yes	Yes	NA	No	Yes
Sepsis	Yes	Yes	Yes	Yes	Yes	Yes	Yes	Yes	Yes	No
Dialysis	Yes	No	No	No	No	No	No	No	No	No
CS	Yes	No	Yes	Yes	Yes	Yes	Yes	0	Yes	Yes
AG	Yes	No	No	No	No	Yes	No	No	Yes	No
Piritramide mg	666	1,832	103	60	72	280	764	200	1032	0
Propofol mg	14,160	27,229	0	15	3,460	0	17,027	603	15,200	0
Vasopressors	Yes	No	Yes	Yes	Yes	Yes	Yes	Yes	Yes	No
NMBA	No	No	Yes	No	No	Yes	Yes	Yes	Yes	No
Total duration of MV hours	555	359	232	49	110	127	700	174	505	65
BIPAP hours	0	232	90	23	0	8	155	53	306	0
PSV hours	280	127	109	9	110	73	504	107	121	14
IPPV hours	275	0	33	17	0	46	41	14	78	51
PEEP at measurement	5	5	5	5	5	5	7	7	7	5

For patients measured on more than one occasion, all data are given for the first measurement. All data concerning dose of medication and duration of mechanical ventilation concern cumulative data from admission to the ICU up to the time of measurement. AG, aminoglycoside; APACHE II, Acute Physiology and Chronic Health Evaluation II; BIPAP, bilevel positive airway pressure; CS, corticosteroids; IPPV, intermittent positive-pressure ventilation; MV, mechanical ventilation; NMBA, neuromuscular blocking agents; PEEP, positive end-expiratory pressure; PSV, pressure-support ventilation; TwPdi, twitch transdiaphragmatic pressure.

### Twitch pressures and repeatability

Mean values of TwPdi, TwPabd, and TwPes in the total case series were calculated by using the mean value for the seven patients that were measured on more than one occasion. The TwPdi was 11.5 ± 3.9 cm H_2_O (mean ± SD); the TwPabd was 5.8 ± 2.3 cm H_2_O (mean ± SD); and the TwPes was 6.7 ± 2.8 cm H_2_O (mean ± SD). The within-occasion coefficient of variation was 7.25% and was calculated by using all accepted signals for all measurements (Table 2). No data are available for patient 3 in this table, as analysis revealed only one acceptable tracing for this patient. The mean between-occasion coefficient of variation was calculated by using the data of patients measured on at least two occasions and was 9.7% (Table 3).

**Table 2:** Within-occasion coefficient of variation for all measurements

Patient number	Measurement occasion	**Pdi (cm H**_2_**O)**	Coefficient of variation (%)
1	M1	12.1	5.6
1	M2	12.4	6.7
2	M1	8.3	3.9
4	M1	12.9	12.2
4	M2	10.4	11.1
5	M1	9.1	9.2
5	M2	8.3	8.8
6	M1	15.4	8.1
6	M2	15.8	8
7	M1	8.4	12.8
7	M2	10.6	2.2
8	M1	9.2	5.2
9	M1	8.9	19.4
9	M2	11.2	1.2
10	M1	18.7	6.0
10	M2	21.9	4.1
10	M3	18.9	2.6
10	M4	22.3	3.4
Mean			7.25

Pdi, transdiaphragmatic pressure.

**Table 3:** Between-occasion coefficient of variation for patients receiving measurements on at least two different occasions

Patient	**Mean value Pdi (cm H**_2_**O)**	Number of measurements	Time span between first and last measurement (hours)	Coefficient of variation (%)
1	12.2	2	24	1.9
4	11.7	2	24	15.5
5	8.7	2	48	6.5
6	15.6	2	48	2.0
7	9.5	2	120	16.6
9	10.0	2	24	16.3
10	20.5	4	72	9.3
Mean	12.6	2,3	51.4	9,7

Pdi, transdiaphragmatic pressure.

### Relation between TwPdi and duration of mechanical ventilation

**Figure 3:**
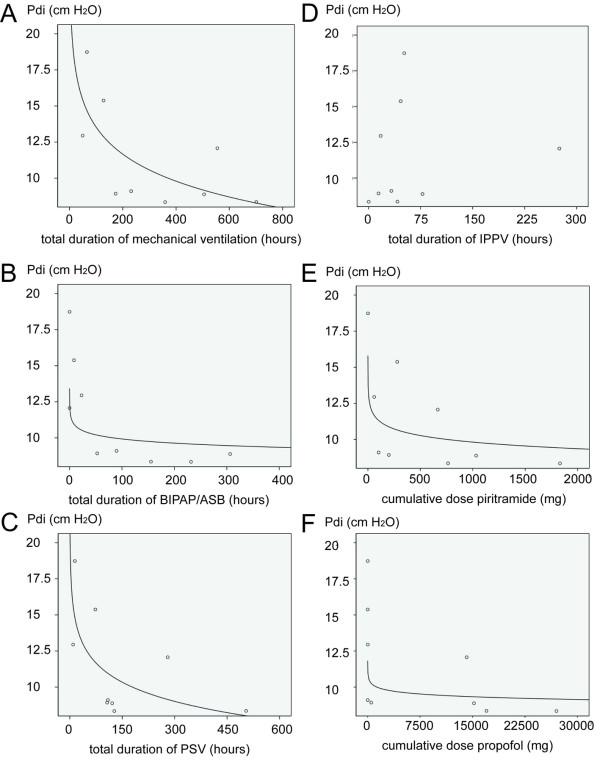
**Regression analysis for TwPdi and total duration of mechanical ventilation; cumulative duration of BIPAP/ASB, PSV, and IPPV at the time of measurement; and cumulative dose of piritramide and propofol**. All data concern values up to the time of measurement of TwPdi. TwPdi, twitch transdiaphragmatic pressure; BIPAP/ASB, bilevel positive airway pressure with assisted spontaneous breathing; PSV, pressure-support ventilation; IPPV, intermittent positive-pressure ventilation.

Regression analysis, excluding the patient with unilateral diaphragm paralysis after surgery, suggested a logarithmic relation between the duration of mechanical ventilation and TwPdi (see Figure 3), with *P *= 0.038 and R = 0.69. To examine whether this effect might be due to other interfering variables, such as sedation or mode of mechanical ventilation, we also performed simple regression analysis for these variables. This suggested a logarithmic relation between TwPdi and the cumulative dose of piritramide up to the time of measurement (R = 0.79; *P *= 0.01); cumulative dose of propofol (R = 0.66; *P *= 0.05); duration of bilevel positive-pressure ventilation (BIPAP/ASB) (R = 0.71; *P *= 0.03); and pressure-support ventilation (PSV) (R = 0.66; *P *= 0.05), but no relation with duration of volume-controlled ventilation (IPPV) (see Figure 3).

## Discussion

In the present study, we evaluated the repeatability of BAMPS Pdi in critically ill mechanically ventilated patients and found results similar to those in healthy volunteers. We also found a logarithmic decline in TwPdi with increasing duration of mechanical ventilation.

Watson *et al*. [19] introduced the use of BAMPS TwPdi as a tool to evaluate diaphragmatic function in critically ill ventilated patients. This group showed that diaphragmatic force measured by using this technique was reduced to about one third of the normal value of healthy controls. These data were confirmed by Laghi *et al*. [20], who also used this method to show that weaning failure was not caused by low-frequency fatigue.

In this study, we measured the TwPdi in critically ill, mechanically ventilated patients on different occasions with a minimum time interval of 24 h to evaluate repeatability of this technique in this setting, which has not been reported. The mean value of TwPdi in our case series was 11.5 ± 3.9 cm H_2_O, which is similar to the previous two case series [19,20] and clearly supports and underscores the fact that these patients have severe respiratory muscle weakness, as values in healthy volunteers have been reported to average from 28 to 38 cm H_2_O [20,22].

Variability between occasions was 9.7%. This figure is very similar to the repeatability in healthy volunteers, which has been reported to be between 6% [23] and 11% [22]. This apparently inherent variability may be due to several factors, such as submaximal nerve stimulation, which occurs in only a minority of measurements, potential changes in lung volume between measurements, changes in thoracoabdominal configuration, and the possibility of twitch potentiation. We did attempt to eliminate all controllable factors, such as positioning of the patient, period of rest after positioning of the balloons and between consecutive stimulations, a constant PEEP level.

We also evaluated the relation between TwPdi obtained and duration of mechanical ventilation at the time of measurement. It is striking that diaphragmatic force appeared to diminish very soon after mechanical ventilation was started in our patients. Regression analysis showed a logarithmic relation. This is the first report suggesting that increased duration of mechanical ventilation is associated with decreased diaphragmatic force, measured with a nonvolitional method, independent of patient cooperation. Previously, Watson *et al*. [19] did not find a clear relation between TwPdi and duration of ICU stay, but no data were explicitly reported concerning duration of mechanical ventilation. Laghi *et al*. [20] did not find a significant difference between TwPdi in the weaning-success group, on average ventilated for 11.5 days, and the weaning-failure group, ventilated for 41.5 days. Although no data for each individual are presented, the averages suggest no major changes in Pdi over a period of about 30 days. The observed association between TwPdi and duration of mechanical ventilation suggests that a major decrease in TwPdi may occur very early, in the first days of mechanical ventilation.

Our findings are compatible with the concept of ventilator-induced diaphragmatic dysfunction, but cannot confirm any causal relation with mechanical ventilation *per se*. Diaphragm unloading and inactivity may be a prime trigger in VIDD, as animal experiments show that assisted mechanical ventilation with a very low back-up rate [24] and intermittent spontaneous breathing [25] attenuates the effect of controlled mechanical ventilation. Therefore, we also looked at the relation between TwPdi and various modes of mechanical ventilation used in our patients. We also found a logarithmic relation between TwPdi and time on BIPAP/assist, as well as time on PSV, but no relation with time on IPPV. It appeared that patients on BIPAP/assist, although they could trigger the ventilator in this mode, did so only with a mean of 0.5 breaths per minute, which actually classifies BIPAP/assist time as full-control mechanical ventilation. When analyzing data on IPPV, we found no relation with TwPdi. If durations on both modes are combined as a measure of full-control mechanical ventilation, this seems to be due to one outlier, patient number 1, who had a fairly preserved TwPdi despite a long duration on IPPV. The number of hours on PSV also is correlated with TwPdi. Although patients on PSV are triggering the ventilator, it may be that diaphragmatic activity is still limited in this mode [26]. The duration of PSV also was highly correlated with total duration of mechanical ventilation, which may explain this finding.

We also examined the relation between TwPdi and the cumulative dose of analgesics/sedation that patients received. Attempts to minimize the amounts of sedatives resulted in reduction in the duration of mechanical ventilation [27]. We daily evaluated patients and titrated sedatives to the lowest dose needed to maintain a SAS score of 3 to 4. We again found a logarithmic relation between TwPdi and the total dose of piritramide as well as propofol that patients had received at the time of measurement. The current data cannot discriminate whether the duration of mechanical ventilation itself or the dose of analgesics/sedatives is responsible for the lower TwPdi values, nor whether other cofactors may have interfered. It is of particular interest that, in contrast with the two previous case series [19,20], all but one patient in our study had from sepsis at some time between the start of mechanical ventilation and the measurement of TwPdi (sepsis incidence in the Watson study, five of 33 patients; in the Laghi study, five of 19 patients). Sepsis is a known risk factor for developing muscle weakness in the ICU and was recently specifically linked to respiratory muscle weakness [2]. This difference may have contributed to our findings and may even be specific for the situation and explain apparently contrasting findings with previous data. As our data set is limited, we cannot exclude that also other factors, such as treatment with corticosteroids and glycemic control, may have contributed.

### Limitations

This is the third case series reporting diaphragmatic force in critically ill patients by using BAMPS. Although the technique appears to have adequate repeatability to discriminate between moderately and severely reduced diaphragmatic force, some variability remains present and should be taken into account when interpreting a single value. This variability seems to be inherent to the method itself, as it also is present in healthy volunteers. In critically ill patients, changes of end-expiratory lung volume over time and between measurements are of particular concern. We attempted to minimize the potential impact by measuring patients at the same level of PEEP on different occasions and checked the flow curve to determine the end-expiration. We did not measure or correct for intrinsic PEEP. We cannot therefore exclude the possibility that changes may have occurred in end-expiratory lung volume between two measurements. As repeatability is very similar to that outside the ICU, the impact seems to have been limited.

We established a decrease of Pdi BAMPS with increasing duration of mechanical ventilation. It is therefore questionable to what extent repeatability of BAMPS Pdi between different occasions can actually be measured in critically ill patients. As we anticipated this possibility, we aimed at measuring patients in time intervals as close together as possible. We did not systematically find lower values on the second occasion than on the first occasion.

The technique is highly sophisticated, fairly invasive, and requires patients to be stable. For these reasons, the technique is currently limited in use to a small subgroup of critically ill patients. As currently no direct therapeutic measures are available to improve patients' respiratory muscle force, the technique only can be offered to patients and their relatives merely for research purposes. Obtaining informed consent for a relatively invasive procedure is therefore not obvious, especially in the early hours and days of admission to the intensive care unit, during which the prognosis of the patient is often unclear and the psychological burden for the relatives is high. Positioning the balloon catheters in patients who are not conscious is often very difficult, as these patients are not able to swallow, and catheters are very flexible and prone to curve in the nasopharynx, as well as in the esophagus. For these reasons, obtaining a large dataset of measurement is very difficult. The limited number of patients implies that we could not perform a stepwise regression analysis to determine the best predictor(s) of TwPdi. The relations that we have established are based on a small dataset, so the results may have been affected by other confounders and must be confirmed.

## Conclusions

We showed that BAMPS TwPdi, which is a nonvolitional method for measuring diaphragmatic force, has acceptable repeatability in critically ill mechanically ventilated patients; this is comparable to the repeatability in healthy volunteers. We observed a logarithmic decline of TwPdi with increased duration of mechanical ventilation. This finding is compatible with the concept of ventilator-induced diaphragmatic dysfunction, but cannot prove the concept. It may be that this observation is due to the use of analgesics and sedatives, or even due to other cofactors, such as sepsis, as the dataset is limited. The technique of BAMPS TwPdi remains a method that is not applicable to a large population of patients because of the fairly invasive nature, technical difficulties, and limitations concerning patients' condition and tolerance.

## Key messages

• Critically ill, mechanically ventilated patients demonstrate profound diaphragm weakness, as assessed by TwPdi, an objective, nonvolitional measure of respiratory muscle strength.

• Duration of mechanical ventilation is associated with a logarithmic decline in diaphragmatic force. Whether this relation is causal or due to other confounders is still unclear.

## Abbreviations

AG: aminoglycosides; APACHE II: Acute Physiology and Chronic Health Evaluation II; ASB: assisted spontaneous breathing; BAMPS: bilateral anterior magnetic phrenic nerve stimulation; BIPAP: bilevel positive airway pressure; CMV: controlled mechanical ventilation; CS: corticosteroids; ICU: intensive care unit; IPPV: intermittent positive-pressure ventilation; MV: mechanical ventilation; NMBAs: neuromuscular blocking agents; PEEP: positive end-expiratory pressure; PSV: pressure-support ventilation; SAS: sedation-agitation scale; TwPdi: twitch transdiaphragmatic pressure; TwPtr: twitch tracheal pressure; TwPabd: twitch abdominal pressure; TwPoes: twitch esophageal pressure; VIDD: ventilator-induced diaphragmatic dysfunction.

## Competing interests

The authors declare that they have no competing interests.

## Authors' contributions

GH drafted the protocol, performed the measurements, analyzed the pressure tracings, obtained patient data, and drafted the manuscript. AA assisted in performing the measurements, obtained patient data, and revised the manuscript. DT assisted in performing the measurements and revised the manuscript. MD had a major impact on the interpretation of data and critical appraisal of the manuscript. GG-R assisted in performing the measurements, obtaining patient data, and had a major impact on the interpretation of data and critical appraisal of the manuscript.
